# Potential Yield Increase of Hybrid Rice at Five Locations in Southern China

**DOI:** 10.1186/s12284-016-0085-6

**Published:** 2016-03-17

**Authors:** Peng Jiang, Xiaobing Xie, Min Huang, Xuefeng Zhou, Ruichun Zhang, Jiana Chen, Dandan Wu, Bing Xia, Hong Xiong, Fuxian Xu, Yingbin Zou

**Affiliations:** Rice and Sorghum Research Institute, Sichuan Academy of Agricultural Sciences/Key Laboratory of Southwest Rice Biology and Genetic Breeding, Ministry of Agriculture, Deyang, 618000 China; Southern Regional Collaborative Innovation Center for Grain and Oil Crops (CICGO), Hunan Agricultural University, Changsha, 410128 China; Luzhou Branch of National Rice Improvement Center, Luzhou, 646100 China

**Keywords:** Grain yield, Hybrid rice, Inbred rice, Yield superiority

## Abstract

**Background:**

A number of field studies have demonstrated that the yield potential of hybrid rice cultivars is higher than that of inbred cultivars, although the magnitude of difference between hybrid and inbred cultivars at different yield levels has not been described. The objective of this study is to compare the yield increase potential at different yield levels between hybrid and conventional rice. Ten field experiments were conducted at five locations in southern China in 2012 and 2013. At each location, two hybrid and two inbred cultivars were grown at three N levels: high (225 kg/hm^2^), moderate (161–191 kg/hm^2^) and the control, zero N (0 kg/hm^2^).

**Results:**

Hybrid rice yielded approximately 8 % more grain than did inbred cultivars in Huaiji, Binyang and Haikou; approximately 7 % more in Changsha; and approximately 19 % more in Xingyi. The high grain yields observed for hybrid rice cultivars were attributed to high grain weight and biomass accumulation at maturity. On average, rice yields were approximately 6.0–7.5 t ha^−1^ (medium yield) in Huaiji, Binyang and Haikou; approximately 9.0 t ha^−1^ in Changsha (high yield); and approximately 12.0 t ha^−1^ (super high yield) in Xingyi. The yield gaps among Huaiji, Binyang and Haikou and Changsha were attributed to the differences in spikelets m^−2^ and biomass production, whereas the yield gap between Changsha and Xingyi was caused by the differences in grain-filling percentage, grain weight and harvest index. The differences in biomass production among sites were primarily due to variation in crop growth rate induced by varied temperatures and accumulative solar radiation.

**Conclusions:**

The yield superiority of hybrid rice was relatively small in comparison with that of inbred cultivars at medium and high yield levels, but the difference was large at super high yield levels. Improving rice yields from medium to high should focus on spikelets m^−2^ and biomass, whereas further improvement to super high level should emphasize on grain-filling percentage, grain weight and harvest index. Favorable environmental conditions are essential for high yields in hybrid rice.

## Background

Rice is a staple food for more than half of the world’s population. Rapid population growth and economic development have imposed growing pressure upon increased food production (Zhang 2007), and a rice yield increase of more than 1.2 % per year will be required in the next decade (Normile 2008). At the same time, paddy area has been decreasing because of expanding construction land area and soil degradation (Jiang et al. 2002). Therefore, most of the future increase in rice production must come from increased rice yields per unit of land area rather than from expanding planting areas. Accordingly, improving rice yield potential has been the main objective of breeders and cultivators in many countries. Rice yield increased rapidly as the result of the development of hybrid rice (Yuan et al. 1994) and combining an ideotype approach with the use of intersubspecific heterosis (Yuan 2001). Peng et al. (1999) reported that potential hybrid rice yields have increased by 9 % over the best inbred cultivars in tropical irrigated lowlands. Similarly, a number of field studies have reported that the grain yield of super hybrid rice was higher than that of inbred rice by more than 10 % (Zhang et al. 2009; Huang et al. 2010). These results show that the higher grain yields of hybrid rice cultivars were associated with large sink size (spikelets per m^2^), which resulted from a large panicle (spikelet number of per panicle).

Recently, a number of field studies have shown that the yield potential of rice could be over 13 t ha^−1^ at some sites, for example, Taoyuan, Yunnan Province, China (Ying et al. 1998; Katsura et al. 2008), which had more twice the yield of the check site yield. Reichardt et al. (2001) and Witt et al. (2001) presented evidence that confirmed a greater role of the environment in rice yield. Katsura et al. (2008) and Li et al. (2009) found that intense incident solar radiation coupled with low nighttime temperatures was the key environmental factor for the high grain yield of irrigated rice in Taoyuan. These results indicated that the environment plays an important role in rice yield. However, these previous studies primarily focused on the differences in grain yield between a high-yielding site and a check site; little attention, however, has been paid to the magnitude of differences in grain yield between hybrid rice and inbred cultivars at different yield levels. Jiang et al. (2015) stated that the magnitude of differences in grain yield between hybrid rice and inbred cultivars depended on their environments. It is very important to clarify the environmental effects on the yield gap between hybrid rice and inbred cultivars as well as the physiological traits of hybrid rice cultivars that lead to their high productivity. The previous studies on the yields and yield attributes of hybrid rice cultivars were conducted in subtropical environments in comparison with inbred cultivars. The differences between different sites in tropical and subtropical environments have been poorly studied.

In our present study, ten field experiments were conducted with four rice cultivars at five sites in China in two years. The objectives of this study were to compare the rice yield differences between hybrid rice and inbred cultivars at different yield levels. Special attention was paid to the yield components and the physiological traits responsible for the magnitude of differences in grain yield between hybrid rice and inbred cultivars at different yield levels. Attention was also paid to the nitrogen responses of hybrid rice and inbred cultivars in different ecological environments.

## Results

### Grain Yield and Yield Components

Averaged across four cultivars, three N treatments and two years, the grain yields were 6.29–7.47 t ha^−1^ in Huaiji, Binyang and Haikou, 9.20 t ha^−1^ in Changsha, and 11.80 t ha^−1^ in Xingyi (Table 1). The differences in grain yield among the cultivars were significant in all ten experiments, although the magnitude of the differences in grain yield between the hybrid and inbred rice varied by site. Averaged across three N rates and two years, the hybrid rice had higher grain yields than did the inbred cultivars by 4.7–10.9 % in Huaiji, Binyang and Haikou; by 6.7 % in Changsha; and by 18.6 % in Xingyi. The yield gaps between the hybrid rice and the inbred cultivars were larger in high-yield cropping environments. N application had significant effects on grain yield in all experiments. The average grain yields under N1 and N2 were 9.03 and 9.11 t ha^−1^, respectively, which were approximately 30 % higher than the yields under N3. The interactive effect between N treatment and cultivar on grain yield was significant only in three of the ten experiments. The mean data across the three N treatments are presented in subsequent tables.

**Table 1:** Grain yield (t ha^−1^) of 4 rice cultivars grown under 3 N treatments at 5 locations in 2012 and 2013

Cultivar	Huaiji				Binyang				Haikou				Changsha				Xingyi			
	N1	N2	N3	Mean	N1	N2	N3	Mean	N1	N2	N3	Mean	N1	N2	N3	Mean	N1	N2	N3	Mean
2012																				
Liangyoupeijiu	7.17	6.86	4.15	6.06	7.79	7.88	6.75	7.47	9.68	8.85	6.93	8.49	9.33	10.48	8.67	9.49	13.18	13.20	10.93	12.44
Y-liangyou 1	7.67	7.19	4.41	6.42	7.13	7.25	5.66	6.68	9.48	9.15	6.61	8.41	9.84	9.88	8.81	9.51	12.91	13.68	10.58	12.39
Yuxiangyouzhan	6.80	6.77	4.14	5.90	7.19	6.72	5.03	6.31	8.90	8.73	6.98	8.20	8.83	9.49	8.19	8.84	10.68	10.78	9.07	10.18
Huanghuazhan	6.95	7.05	4.13	6.04	7.41	7.83	5.28	6.84	9.00	8.47	6.74	8.07	9.09	9.44	8.32	8.95	10.77	11.08	9.22	10.36
Mean	7.15	6.97	4.21	6.11 e	7.38	7.42	5.68	6.83 d	9.26	8.80	6.81	8.29 c	9.27	9.82	8.50	9.20 b	11.89	12.19	9.95	11.34 a
Analysis of variance																				
genotype (G)	^b^				^b^				^b^				^b^				^b^			
N treatment (N)	^a^				^b^				^b^				^b^				^b^			
G × N	ns				ns				^a^				ns				ns			
2013																				
Liangyoupeijiu	7.00	6.98	5.47	6.48	8.79	8.96	6.24	8.00	7.73	7.15	5.91	6.93	10.07	10.19	8.31	9.52	13.21	13.88	11.85	12.98
Y-liangyou 1	7.15	7.66	5.53	6.78	8.85	9.08	5.67	7.87	7.25	7.61	6.19	7.02	9.97	10.82	7.58	9.46	14.09	13.90	12.16	13.38
Yuxiangyouzhan	7.46	6.58	4.91	6.32	7.97	8.05	5.24	7.09	6.51	6.86	5.16	6.18	9.17	9.46	7.21	8.61	11.95	12.13	10.56	11.55
Huanghuazhan	6.89	7.12	4.99	6.33	8.20	7.77	4.43	6.80	6.76	7.17	5.32	6.42	10.53	10.46	6.61	9.20	11.73	11.77	9.79	11.10
Mean	7.13	7.09	5.23	6.48 d	8.45	8.46	5.40	7.44 c	7.06	7.20	5.65	6.64 d	9.94	10.23	7.43	9.20 b	12.75	12.92	11.09	12.25 a
Analysis of variance																				
Genotype (g)	^b^				^b^				^b^				^b^				^b^			
N treatment (N)	^a^				^b^				^b^				^b^				^b^			
G × N	^b^				^a^				ns				ns				ns			

Within the row for each location, means followed by the same letters are not significantly different according to LSD at *P* = 0.05

^a^Significant at the 0.05 level based on analysis of variance

^b^Significant at the 0.01 level based on analysis of variance. ns denotes non-significance based on analysis of variance

Averaged across four cultivars and two years, panicles m^−2^, spikelets per panicle and spikelets m^−2^ in Huaiji, Binyang and Haikou were significantly lower than those in Changsha by 16–26 %, 14–25 % and 35–40 %, respectively, whereas the differences in spikelet filling percentage and grain weight were relatively small or inconsistent (Tables 2 and 3). Although Changsha had approximately 7 % more spikelet panicles^−1^ than Xingyi, there were 12 % fewer panicles m^−2^ in Changsha than in Xingyi. Consequently, there were 5 % fewer spikelets m^−2^ in Changsha than in Xingyi. Both spikelet filling percentage and grain weight were lower in Changsha than in Xingyi by approximately 8 %. Although Huanghuazhan recorded the most panicles per m^2^ and Yuxiangyouzhan produced the most spikelets per panicle, the differences in spikelets per m^2^ between hybrid rice and inbred cultivars were relatively small or inconsistent. Hybrid rice had a higher grain weight than did inbred cultivars by 18.5–22.5 % in Huaiji, Binyang and Haikou; by 21.1 % in Changsha; and by 19.1 % in Xingyi. The differences in spikelet filling percentage between hybrid rice and inbred cultivars were small or inconsistent.

**Table 2:** Yield components of 4 rice cultivars grown under 3 N treatments at 5 locations in 2012. Data are averaged across 3 N treatments

Cultivar	Panicles m^−2^	Spikelets panicle^−1^	Spikelets m^−2^ (× 10^3^)	Spikelet filling (%)	Grain weight (mg)
Huaiji					
Liangyoupeijiu	186.6	161.4	30.4	74.3	23.6
Y-liangyou 1	197.3	147.4	29.5	79.6	24.0
Yuxiangyouzhan	176.5	180.2	31.9	78.6	19.8
Huanghuazhan	211.7	145.5	31.0	82.9	20.0
Mean	193.1 d	158.6 d	30.7 d	78.8 c	21.8 ab
LSD (0.05)	17.2	8.7	1.7	3.6	0.4
Binyang					
Liangyoupeijiu	204.1	147.2	30.1	81.8	24.3
Y-liangyou 1	205.2	139.1	28.8	75.4	24.3
Yuxiangyouzhan	167.5	179.0	29.9	84.3	21.1
Huanghuazhan	217.1	142.4	30.8	85.9	21.3
Mean	198.5 cd	151.9 d	29.9 d	81.8 b	22.8 a
LSD (0.05)	14.1	7.8	2.0	2.9	0.3
Haikou					
Liangyoupeijiu	227.1	151.0	34.4	85.9	25.3
Y-liangyou 1	214.8	157.4	34.1	83.8	25.1
Yuxiangyouzhan	194.9	211.9	41.3	88.5	20.2
Huanghuazhan	219.6	173.4	38.1	89.1	20.5
Mean	214.1 c	173.4 c	37.0 c	86.8 a	22.8 a
LSD (0.05)	10.9	7.5	2.0	2.5	0.2
Changsha					
Liangyoupeijiu	219.7	240.3	52.7	74.3	23.4
Y-liangyou 1	231.3	205.7	47.7	84.1	23.6
Yuxiangyouzhan	218.4	243.7	53.0	83.2	19.7
Huanghuazhan	287.1	171.2	48.8	84.0	20.0
Mean	239.1 b	215.2 a	50.5 b	81.4 bc	21.7 b
LSD (0.05)	8.7	9.5	3.1	4.0	0.3
Xingyi					
Liangyoupeijiu	277.6	188.3	52.5	83.4	24.6
Y-liangyou 1	284.9	185.2	52.9	86.3	24.9
Yuxiangyouzhan	257.9	230.9	59.5	85.6	20.0
Huanghuazhan	317.0	181.4	57.5	86.0	20.9
Mean	284.3 a	196.4 b	55.6 a	85.3 a	22.6 ab
LSD (0.05)	16.5	8.4	4.3	2.7	0.5

Within the column for each location, means followed by the same letters are not significantly different according to LSD at *P* = 0.05

LSD values are for the comparison of cultivars for each parameter at each location

**Table 3:** Yield components of 4 rice cultivars grown under 3 N treatments in 5 locations at 2013. Data are averaged across 3 N treatments

Cultivar	Panicles m^−2^	Spikelets panicle^−1^	Spikelets m^−2^ (× 10^3^)	Spikelet filling (%)	Grain weight (mg)
Huaiji					
Liangyoupeijiu	171.5	217.0	37.5	65.7	23.4
Y-liangyou 1	195.3	195.6	38.7	69.7	23.4
Yuxiangyouzhan	175.7	226.1	39.6	71.5	19.6
Huanghuazhan	197.4	184.3	36.2	82.0	19.6
Mean	184.9 c	205.7 a	38.0 b	72.2 d	21.5 b
LSD (0.05)	16.2	14.9	3.9	4.1	0.4
Binyang					
Liangyoupeijiu	206.4	171.5	35.5	80.2	24.8
Y-liangyou 1	201.6	155.3	31.6	82.2	25.3
Yuxiangyouzhan	196.7	183.7	35.9	83.8	20.5
Huanghuazhan	220.8	153.0	33.8	83.4	20.4
Mean	206.4 b	165.9 b	34.2 c	82.4 b	22.7 a
LSD (0.05)	14.1	11.1	2.7	2.9	0.3
Haikou					
Liangyoupeijiu	212.8	152.5	32.4	77.4	24.2
Y-liangyou 1	211.6	154.1	33.1	74.4	25.2
Yuxiangyouzhan	208.4	156.9	32.4	90.7	20.0
Huanghuazhan	228.3	144.2	32.7	90.5	20.8
Mean	215.3 b	151.9 c	32.6 c	83.3 ab	22.6 a
LSD (0.05)	15.8	11.2	2.4	3.3	0.3
Changsha					
Liangyoupeijiu	252.3	230.6	58.3	70.5	22.6
Y-liangyou 1	256.7	185.0	47.6	83.8	22.7
Yuxiangyouzhan	258.8	229.1	59.4	75.4	18.6
Huanghuazhan	315.2	192.5	59.5	77.5	17.9
Mean	270.7 a	209.3 a	56.2 a	76.8 c	20.5 c
LSD (0.05)	20.3	16.7	4.1	4.7	0.4
Xingyi					
Liangyoupeijiu	283.6	208.0	58.9	81.4	24.5
Y-liangyou 1	307.2	180.3	55.4	86.1	25.8
Yuxiangyouzhan	242.5	244.0	58.6	89.9	20.6
Huanghuazhan	334.0	164.1	54.0	88.5	22.3
Mean	291.8 a	199.1 a	56.7 a	86.5 a	23.3 a
LSD (0.05)	19.1	16.1	4.2	2.7	0.5

Within the column for each location, means followed by the same letters are not significantly different according to LSD at *P* = 0.05

LSD values are for the comparison of cultivars for each parameter at each location

### Biomass Production and Crop Growth Rates

The average total biomass across four cultivars and two years was 31–39 % lower in Huaiji, Binyang and Haikou than in Changsha, although the differences in the harvest index were relatively small (Table 4). There were significant differences in both total biomass and harvest index between Changsha and Xingyi. The average total biomass and harvest index in Changsha were lower than in Xingyi by 10 and 8 %, respectively. Hybrid cultivars generally had higher total biomass than did inbred cultivars, but the difference in the harvest index was inconsistent.

**Table 4:** Total biomass and harvest index of 4 rice cultivars grown under 3 N treatments at 5 locations in 2012 and 2013. Data are averaged across 3 N treatments

Cultivar	2012		2013	
	Total biomass (g m^−2^)	Harvest index (%)	Total biomass (g m^−2^)	Harvest index (%)
Huaiji				
Liangyoupeijiu	1051.3	50.1	1190.4	48.1
Y-liangyou 1	1048.5	52.9	1305.8	47.4
Yuxiangyouzhan	981.0	50.2	1103.8	49.9
Huanghuazhan	977.9	52.2	1130.6	51.4
Mean	1014.7 d	51.3 c	1182.7 c	49.2 d
LSD (0.05)	66.2	1.34	109.3	2.1
Binyang				
Liangyoupeijiu	1258.8	47.6	1344.4	52.3
Y-liangyou 1	1227.3	42.7	1275.4	51.4
Yuxiangyouzhan	1180.4	45.1	1158.8	53.3
Huanghuazhan	1190.4	47.9	1098.8	52.5
Mean	1214.2 c	45.8 d	1219.4 c	52.4 bc
LSD (0.05)	60.4	1.6	57.1	1.5
Haikou				
Liangyoupeijiu	1366.7	54.7	1186.0	51.3
Y-liangyou 1	1304.3	54.5	1199.9	50.7
Yuxiangyouzhan	1333.7	55.0	1135.5	51.8
Huanghuazhan	1302.2	53.4	1178.0	52.6
Mean	1326.7 c	54.4 b	1174.9 c	51.6 c
LSD (0.05)	47.1	0.9	57.4	1.7
Changsha				
Liangyoupeijiu	1884.1	50.8	1910.4	53.8
Y-liangyou 1	1909.0	52.3	1857.4	55.7
Yuxiangyouzhan	1738.8	53.2	1742.4	50.2
Huanghuazhan	1729.0	50.5	1756.6	51.8
Mean	1815.2 b	51.7 c	1816.7 b	52.9 b
LSD (0.05)	74.4	1.1	94.0	1.6
Xingyi				
Liangyoupeijiu	2061.0	55.3	2060.0	56.8
Y-liangyou 1	2104.7	57.2	2084.5	58.4
Yuxiangyouzhan	1957.1	55.5	1908.8	56.6
Huanghuazhan	2048.0	55.3	1881.5	58.0
Mean	2042.7 a	55.8 a	1983.7 a	57.4 a
LSD (0.05)	85.8	1.3	141.4	1.8

Within the column for each location, means followed by the same letters are not significantly different according to LSD at *P* = 0.05

LSD values are for the comparison of cultivars for each parameter at each location

**Fig. 1:**
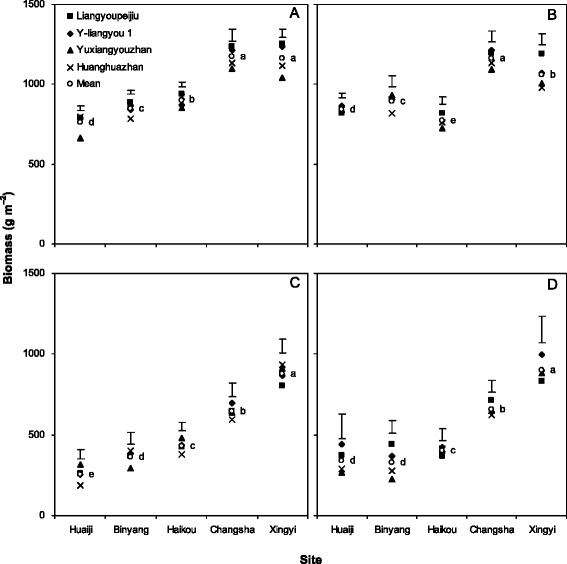
Biomass accumulation from sowing to full heading (**a**, **b**) and from full heading to maturity (**c**, **d**) in 4 rice cultivars at 5 locations in 2012 (**a**, **c**) and 2013 (**b**, **d**). Data are averaged across 3 N treatments. Means followed by the same letters are not significant at the 0.05 level. Error bars are for the comparison of cultivars at each location

**Fig. 2:**
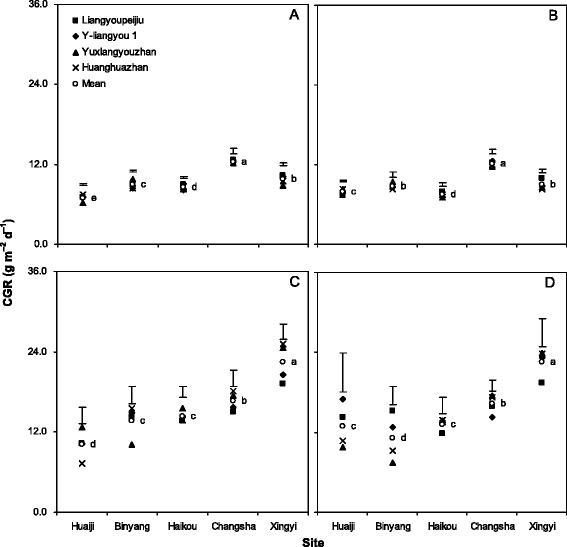
Crop growth rate (CGR) from sowing to full heading (**a**, **b**) and from full heading to maturity (**c**, **d**) of 4 rice cultivars at 5 locations in 2012 (**a**, **c**) and 2013 (**b**, **d**). Data are averaged across 3 N treatments. Means followed by the same letters are not significant at the 0.05 level. Error bars are for the comparison of cultivars in each location

Biomass production, averaged across four cultivars and two years, was lower in Huaiji, Binyang and Haikou than in Changsha by 25–31 % from SO to HD and by 36–54 % from HD to MA (Fig. 1). Changsha produced similar or higher biomass production than Xingyi from SO to HD, whereas from HD to MA, biomass production in Changsha was lower than that in Xingyi by an average of 27 %. Higher biomass production of hybrid cultivars from SO to HD than inbred cultivars was observed in Changsha and Xingyi but not in Huaiji, Binyang or Haikou. There were inconsistent differences in biomass accumulation from HD to MA between hybrid and inbred cultivars. The average crop growth rate (CGR) from SO to HD and HD to MA was, respectively, 28–39 % and 16–29 % lower in Huaiji, Binyang and Haikou than in Changsha (Fig. 2). The average CGR was 31 % higher from SO to HD but 27 % lower from HD to MA in Changsha than in Xingyi. There were inconsistent differences in CGR between hybrid and inbred cultivars.

Averaged across four cultivars and two years, growth durations were 4–13 d longer from SO to HD but 10–15 d shorter from HD to MA in Huaiji, Binyang and Haikou than in Changsha (Table 5). Changsha had an approximately 25 d shorter growth duration than did Xingyi from SO to HD, whereas the growth duration from HD to MA was nearly the same at the two sites. Longer growth duration was generally observed in the hybrid rice (Liangyoupeijiu and Yliangyou-1) than in the inbred cultivars (Huanghuazhan and Yuxiangyouzhan).

**Table 5:** Growth duration, and temperature and accumulative solar radiation during growing season of 4 rice cultivars grown at 5 sites in 2012 and 2013

Year	Site	Cultivar	Growth duration (d)		Mean maximum temperature (°C)		Mean minimum temperature (°C)		Accumulative solar radiation (MJ m^−2^)	
			SO-HD^a^	HD-MA	SO-HD	HD-MA	SO-HD	HD-MA	SO-HD	HD-MA
2012	Huaiji	Liangyoupeijiu	112	25	28.0	34.1	20.6	24.8	2162	575
		Y liangyou 1	112	25	28.0	34.1	20.6	24.8	2162	575
		Yuxiangyouzhan	106	25	27.9	32.9	20.3	24.8	2056	538
		Huanghuazhan	106	25	27.9	32.9	20.3	24.8	2056	538
		Mean	109	25	27.9	33.5	20.4	24.8	2109	557
	Binyang	Liangyoupeijiu	99	26	29.5	33.6	22.6	26.2	1871	530
		Y liangyou 1	99	26	29.5	33.6	22.6	26.2	1871	530
		Yuxiangyouzhan	90	29	29.3	32.6	22.3	26.0	1710	563
		Huanghuazhan	93	26	29.4	32.8	22.4	26.1	1767	507
		Mean	95	27	29.4	33.1	22.5	26.1	1805	533
	Haikou	Liangyoupeijiu	105	31	25.9	34.6	18.8	24.7	1722	722
		Y liangyou 1	106	30	25.9	34.6	18.8	24.8	1749	699
		Yuxiangyouzhan	103	30	25.7	34.7	18.7	24.6	1679	705
		Huanghuazhan	103	30	25.7	34.7	18.7	24.6	1679	705
		Mean	104	30	25.8	34.7	18.7	24.7	1707	708
	Changsha	Liangyoupeijiu	98	44	31.7	31.0	24.3	22.8	1999	823
		Y liangyou 1	98	44	31.7	31.0	24.3	22.8	1999	823
		Yuxiangyouzhan	89	37	31.6	33.0	24.1	25.0	1825	725
		Huanghuazhan	93	33	31.7	32.9	24.2	24.8	1907	644
		Mean	95	40	31.7	32.0	24.3	23.8	1933	754
	Xingyi	Liangyoupeijiu	121	42	26.6	27.8	18.4	19.4	2541	861
		Y liangyou 1	121	42	26.6	27.8	18.4	19.4	2541	861
		Yuxiangyouzhan	118	37	26.5	27.9	18.3	19.5	2478	766
		Huanghuazhan	118	37	26.5	27.9	18.3	19.5	2478	766
		Mean	120	40	26.6	27.8	18.4	19.5	2510	814
2013	Huaiji	Liangyoupeijiu	111	26	27.5	33.1	20.1	24.5	2142	577
		Y liangyou 1	111	26	27.5	33.1	20.1	24.5	2142	577
		Yuxiangyouzhan	101	27	26.9	33.5	19.6	24.8	1919	605
		Huanghuazhan	101	27	26.9	33.5	19.6	24.8	1919	605
		Mean	106	27	27.2	33.3	19.8	24.6	2031	591
	Binyang	Liangyoupeijiu	105	29	28.4	33.4	21.8	26.0	1932	595
		Y liangyou 1	105	29	28.4	33.4	21.8	26.0	1932	595
		Yuxiangyouzhan	99	30	28.1	33.6	21.5	26.2	1812	615
		Huanghuazhan	99	30	28.1	33.6	21.5	26.2	1812	615
		Mean	102	30	28.3	33.5	21.6	26.1	1872	605
	Haikou	Liangyoupeijiu	103	31	27.9	34.0	20.1	24.5	1803	701
		Y liangyou 1	103	31	27.9	34.0	20.1	24.5	1803	701
		Yuxiangyouzhan	102	30	27.8	33.8	20.1	24.6	1775	669
		Huanghuazhan	102	30	27.8	33.8	20.1	24.6	1775	669
		Mean	103	31	27.9	33.9	20.1	24.5	1789	685
	Changsha	Liangyoupeijiu	97	45	34.4	30.8	25.3	23.3	2202	783
		Y liangyou 1	97	45	34.4	30.8	25.3	23.3	2202	783
		Yuxiangyouzhan	93	37	34.1	32.4	25.1	25.0	2111	677
		Huanghuazhan	94	36	34.2	32.2	25.2	24.9	2134	652
		Mean	95	41	34.3	31.6	25.2	24.2	2162	724
	Xingyi	Liangyoupeijiu	120	43	27.5	26.5	18.9	18.6	2616	813
		Y liangyou 1	120	43	27.5	26.5	18.9	18.6	2616	813
		Yuxiangyouzhan	117	37	27.5	26.7	18.9	18.7	2562	714
		Huanghuazhan	117	37	27.5	26.7	18.9	18.7	2562	714
		Mean	119	40	27.5	26.6	18.9	18.7	2589	764

^a^SO–HD, from sowing to full heading; HD–MA, from full heading to maturity

### Climatic Factors and Relationship with Grain Yield

**Fig. 3:**
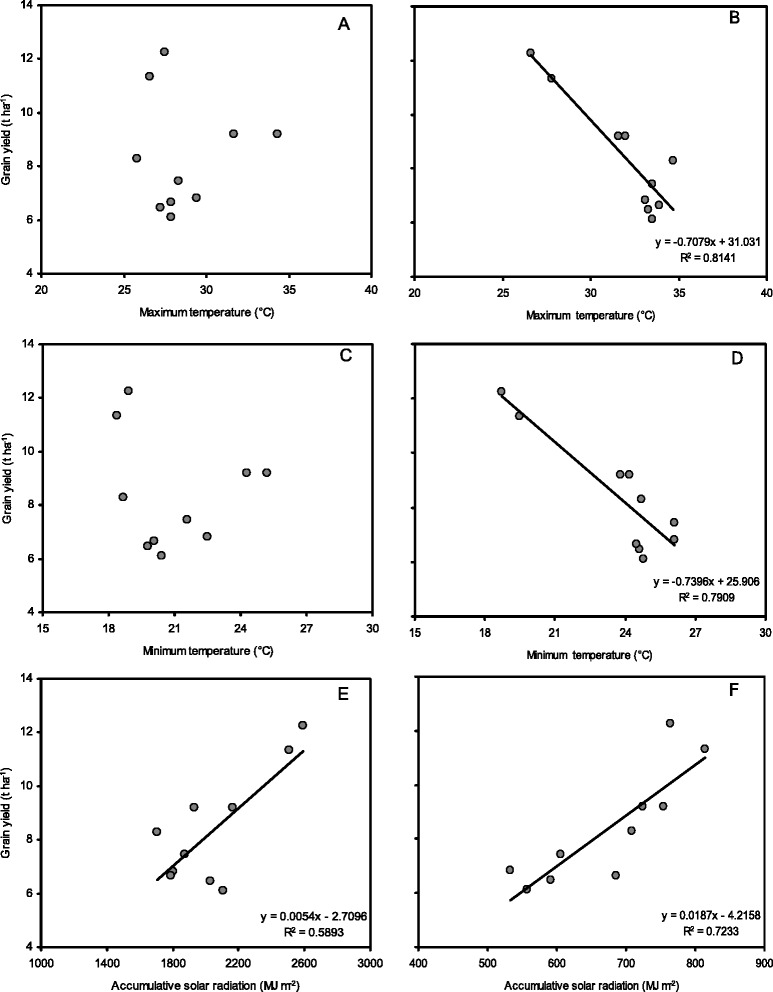
The relationships of grain yield with mean maximum temperature (**a**), mean minimum temperature (**c**), accumulative solar radiation (**e**) from sowing to heading, and mean maximum temperature (**b**), mean minimum temperature (**d**), accumulative solar radiation (**f**) heading to maturity from heading to maturity of 4 rice cultivars at 5 locations in 2012 and 2013. Data are averaged across 3 N treatments and 4 rice cultivars

The mean maximum temperature was lower in Huaiji, Binyang and Haikou than in Changsha from sowing (SO) to heading (HD) by 2.3–7.1 °C in 2012 and 2013, whereas from HD to maturity (MA), the mean maximum temperatures were higher in Huaiji, Binyang and Haikou than in Changsha by 1.1–2.7 °C in 2012 and 2013 (Table 5). Similar but smaller differences were detected for the minimum temperatures. Changsha had higher mean maximum and minimum temperatures than did Xingyi by approximately 6.0 °C from SO to HD and by approximately 5.0 °C from HD to MA, averaged across two years. Accumulative solar radiation from SO to HD and HD to MA in Huaiji, Binyang and Haikou was similar to or lower than that in Changsha. Changsha had lower accumulative solar radiation from SO to HD and HD to MA in than did Xingyi in both years. Hybrid rice had similar or slightly higher maximum and minimum temperatures from SO to HD than did inbred cultivars, whereas from HD to MA, these temperatures were lower for hybrid rice than for inbred cultivars except in Huaiji and Binyang in 2012. Grain yield was negatively related to mean maximum and minimum temperatures from HD to MA but not to those from SO to HD (Fig. 3a–d). There were positive relationship between grain yield and accumulative solar radiations from SO to HD and from HD to MA (Fig. 3e and f), and grain yield was related more closely to accumulative solar radiation from HD to MA than that from SO to HD.

## Discussion

In our present study, hybrid rice out-yielded inbred cultivars at five sites in 2012 and 2013, although the magnitude of differences in grain yields between hybrid cultivars and inbred cultivars varied by site. On average, hybrid rice had higher grain yields than inbred cultivars by approximately 8 % in Huaiji, Binyang and Haikou; approximately 7 % in Changsha; and approximately 19 % in Xingyi. Because of intensive crop management practices, no major abiotic or biotic stresses that caused yield reductions were observed in any of the ten field experiments, and grain yields were approximately 6.0–7.5 t ha^−1^ (medium yield) in Huaiji, Binyang and Haikou; 9.0 t ha^−1^ (high yield) in Changsha; and 12.0 t ha^−1^ (super high yield) in Xingyi. These results indicated that the yield advantage of hybrid rice was less than 8 % in comparison with inbred cultivars at the medium and high yield levels, but it was approximately 19 % in comparison with inbred cultivars at the super high level. These findings suggest that favorable environmental conditions are essential for realizing the high yield potential of hybrid rice. On one hand, Xingyi had, respectively, lower maximum and minimum temperatures during grain filling period than did Huaiji, Binyang, Haikou and Changsha by 4.2–7.3 °C and 4.3–7.4 °C, whereas Xingyi had higher accumulative solar radiation than did the other four sites by 40–281 MJ m^−2^. These results indicates that cool air temperature and high accumulative solar radiation during grain filling period are two key climatic factors for high grain yield of hybrid rice. This was further supported by that there were tight linear relationships between grain yield with mean maximum and minimum temperatures and accumulative solar radiation during grain filling period. On the other hand, soil indigenous nutrient supply is of great importance for rice growing (Shoji et al. 1974). In the present study, soil organic matter, total N and available K contents were higher in Xingyi than in Huaiji, Binyang, Haikou and Changsha (Table 6). Accordingly, grain yield in zero N treatment was higher in Xingyi than in Huaiji, Binyang, Haikou and Changsha (Table 1). More interestingly, the yield difference between hybrid and inbred cultivars in zero N treatment was larger in Xingyi than in Huaiji, Binyang, Haikou and Changsha. This finding suggests that higher fertility soil was partially responsible for the lager yield advantage of hybrid rice in Xingyi than in the other four locations. These results are in agreement with that reported by Jiang et al. (2016), who reported that both soil indigenous and applied N were key factors for improving rice yield from medium (6.5–7.5 t ha^−1^) to high level (9.0 t ha^−1^), while a further improvement to super high yield (12.0 t ha^−1^) soil indigenous N was more important than fertilizer N.

**Table 6:** Soil properties of the experimental fields

Site	pH	Organic matter (g kg^−1^)	Total N (g kg^−1^)	Available P (mg kg^−1^)	Available K (mg kg^−1^)
Huaiji	5.2	33.8	1.7	9.2	77.2
Binyang	5.1	28.9	1.6	34.1	183.8
Haikou	5.9	21.4	1.0	34.8	114.5
Changsha	5.8	27.7	1.6	54.5	63.2
Xingyi	7.9	52.6	2.6	16.5	257.2

The higher grain yields observed for hybrid rice cultivars are attributed to higher grain weight, on average, which was approximately 20 % higher for hybrid rice cultivars than for inbred rice cultivars. Similar result was also observed by Huang et al. (2012), who reported that hybrid varieties had 23 % higher grain weight than inbred cultivars. However, Zhang et al. (2009) and Bueno and Lafarge (2009) reported that there was no consistent difference in grain weight between hybrid and inbred rice cultivars. These suggest that large grain weigh is not a general trait in hybrid rice cultivars, but developing rice cultivars with high grain weight may be a feasible approach to achieve high grain yield. Grain weight is determined not only by grain capacity to receive assimilates but also by the source supplying assimilates to grains (Ntanos and Koutroubas 2002). In our present study, hybrid rice cultivars produced higher biomass than did inbred cultivars except for Haikou in 2012. This result suggests that source capacity is critical to the high grain weight of hybrid rice cultivars. In addition, although hybrid rice cultivars did not show superiority in the harvest index, panicles per m^2^, spikelets per panicle and grain filling, their values were relatively high; thus, the advantage of high biomass production and high grain weight was not offset by other components or attributes of grain yield. The findings indicated that the present strategy to increase rice yield for irrigated rice increased grain weight while maintaining the other yield components.

The high biomass production of hybrid rice cultivars observed for Huaiji, Binyang and Haikou was attributed to long growth duration because the differences in CGR from SO to HD and HD to MA between hybrid rice and inbred cultivars were relatively small or inconsistent. In Changsha and Xingyi, the high CGR from SO to HD and long growth duration from SO to HD and HD to MA were responsible for the high biomass production in hybrid rice cultivars. Our results suggest that long growth duration is a specific trait for hybrid rice cultivars, and hybrid vigor may be not critical for yield difference between hybrid and inbred rice cultivars.

The low grain yields observed for Huaiji, Binyang and Haikou were attributed to small sink size (spikelets m^−2^), which resulted from low panicle number per m^2^ and spikelet number per panicle because the differences in spikelet filling percentages and grain weight between them were relatively small or inconsistent. The importance of sink size in enhancing grain yield has been reported in many studies (Kropff et al. 1994; Ying et al. 1998; Zhang et al. 2009; Ibrahim et al. 2013). However, when Changsha and Xingyi were compared, the differences in sink size, spikelet filling percentage and grain weight were attributed to the yield gap between them. More interesting, Xingyi had a larger sink size than Changsha, though Xingyi consistently had higher grain filling than Changsha. It is probable that high mean maximum and minimum temperatures and low accumulative solar radiation during the grain filling period in Changsha compared with Xingyi reduced the grain filling of four cultivars (Tables 2 and 3). This conclusion is consistent with previous reports by Seshu and Cady (1984) and Kobata et al. (2006) that high night temperatures reduced assimilate supply to the grains during grain filling, especially when followed by low light intensity. These results suggest that improving rice yield from medium (6.0–7.5 t ha^−1^) to high (9.0 t ha^−1^) should focus on sink size and that improvement to a super high level (12.0 t ha^−1^) should rely on simultaneous increases in sink size, spikelet filling percentage and grain weight.

The yield gaps between Huaiji, Binyang and Haikou and Changsha could be explained by the differences in total biomass because the differences in harvest index between them were relatively small. This possibility is in agreement with previous studies (Song et al. 1990; Yamauchi 1994; Horie et al. 1997), which reported that further improvement in rice yield might come from an increase in biomass production rather than in the harvest index. However, in the present study, the higher grain yield in Xingyi was attributed to both higher total biomass and higher harvest index than in Changsha. These results indicate that increasing biomass production is critical for improving rice yield from medium to high levels and improvement to super high yield depends on increasing both biomass production and harvest index. Furthermore, rice grain yield is the function of biomass accumulation after heading and the translocation of biomass accumulated before heading to grains (Yang et al. 2008). Although both are associated with grain yield, Miah et al. (1996) and Laza et al. (2003) stated that translocation of pre-stored reserves should be emphasized more than biomass accumulated during ripening for increasing grain yield, whereas Yang et al. (2008) observed that grain yield was closely associated with biomass accumulated after heading. In the present study, biomass production was higher in Changsha than in Huaiji, Binyang and Haikou both during SO to HD and during HD to MA, but it was higher in Xingyi than in Changsha only during HD to MA. These findings indicate that both biomass accumulation before and after heading are important for improving rice yield from medium to high levels, whereas additional improvement to super high yields should focus on increasing the biomass production after heading.

The higher biomass production during SO to HD in Changsha than in Huaiji, Binyang and Haikou was due to the higher CGR in Changsha because growth duration during SO to HD was shorter in Changsha than at the three sites. The higher biomass accumulation from HD to MA in Changsha than at the three sites was attributed to both the longer growth duration and higher CGR in Changsha. The higher biomass production during HD to MA in Xingyi than in Changsha resulted from the higher CGR in Xingyi because growth duration from HD to MA was comparable between the two sites. These results reveal that longer growth duration is not the only factor that contributes to higher grain yield. CGR is a function of canopy gross photosynthesis and crop respiration (Evans 1993), both of which are influenced by temperature (Loomis and Connor 1992; Akita 1993). In the present study, the higher CGR during SO to HD in Changsha than in Huaiji, Binyang and Haikou was associated with that location’s higher canopy photosynthesis, caused by its higher day temperatures, whereas the higher CGR during HD to MA in Changsha than in Huaiji, Binyang and Haikou as well as in Xingyi than in Changsha was related to lower crop respiration that resulted from lower night temperatures. In addition, the differences in accumulative solar radiation accumulation were partly responsible for the in CGR among the sites.

## Conclusions

Hybrid rice cultivars produced higher grain yields than did inbred cultivars; however, the magnitude of the differences in grain yield between them varied by site. The high grain yields observed for hybrid rice cultivars were attributed to high grain weight and biomass accumulation at maturity. The long growth duration was partially responsible for the high biomass production in hybrid rice cultivars. Rice yield levels were medium (approximately 6.0–7.5 t ha^−1^) in Huaiji, Binyang and Haikou; high in Changsha (approximately 9.0 t ha^−1^); and super high (approximately 12.0 t ha^−1^) in Xingyi. The yield gaps between Huaiji, Binyang and Haikou and Changsha were attributed to the differences in sink (spikelets m^−2^) and biomass production (source), whereas the yield gap between Changsha and Xingyi was caused by the differences in sink, source and flow (grain-filling percentage, grain weight and harvest index). The differences in biomass production among sites were mostly due to variations in crop growth rates induced by varying temperatures and accumulative solar radiation. These results indicated that the yield gap between hybrid rice with inbred cultivars was relatively small at medium and high yield levels but large at the super high yield level. In addition, improving rice yields from medium to high should focus on sink and source, and further improvement to super high yields should emphasize sink, source and flow. In other words, favorable environmental conditions are essential for high hybrid rice yields.

## Methods

### Field Experiments

Field experiments were conducted in fields in the major rice-growing areas of China in 2012 and 2013. The experimental sites were located in Huaiji County (24°03′52″N, 112°03′27″E, 70 m asl) in Guangdong Province, Binyang County (23°09′33″N, 108°52′43″E, 90 m asl) in Guangxi Province, Haikou City (19°45′12″N, 110°11′52″E, 26 m asl) in Hainan Province, Changsha City (28°11′11″N, 113°04′15″E, 45 m asl) in Hunan Province, and Xingyi City (25°07′16″N, 104°55′56″E, 1170 m asl) in Guizhou Province. The properties of the experimental soils are presented in Table 6. The soil tests were based on samples taken from the upper 20 cm of the soil before the rice was transplanted in the first year.

Two super hybrid rice cultivars, Liangyoupeijiu and Yliangyou-1 and two high-yielding inbred cultivars, Yuxiangyouzhan and Huanghuazhan, were used at each site. Liangyoupeijiu is an *indica-japonica* hybrid (Peiai64S × 9311) developed by the Jiangsu Academy of Agricultural Science and released in 1999. Yliangyou-1 is an *indica* hybrid (Y58S × 9311) developed by the Hunan Academy of Agricultural Science and released in 2006. Yuxiangyouzhan and Huanghuazhan are *inidca* inbreds developed by the Guangdong Academy of Agricultural Science and released in 2005. These cultivars have been widely grown by rice farmers in China because of their good yields.

Treatments were arranged in a split-plot design with N treatments as the main plots and the cultivars as subplots. The experiment was replicated three times, and the subplot size was 20 m^2^. The three N treatments were (i) high N (225 kg ha^−1^, N1), (ii) moderate N (161–191 kg ha^−1^, N2) and (iii) zero N, the control (0 kg ha^−1^, N3). For the N1 treatment, 112.5, 45, 45 and 22.5 kg N ha^−1^ were applied at baseline (1 day before transplanting), early tillering (7 days after transplanting), panicle initiation (the first appearance of a differentiated apex), and spikelet differentiation (the appearance of glumous flower primordia at the tips of elongating primary rachis branches), respectively. For N2 treatment, N application timing and rates are given in Table 7.

**Table 7:** Nitrogen application timing and rate in moderate N treatment at each experimental site in 2012 and 2013

Year	Site	N application timing and rate (kg ha^−1^)^a^				Total N application rate (kg ha^−1^)
		Basal	Tillering	Panicle initiation	Spikelet differentiation	
2012	Huaiji	56	60	30	15	161
	Binyang	56	60	45	15	176
	Haikou	56	60	45	0	161
	Changsha	56	60	45	0	161
	Xingyi	56	60	45	0	161
2013	Huaiji	56	60	60	15	191
	Binyang	56	60	60	0	176
	Haikou	56	60	45	15	176
	Changsha	56	60	45	0	161
	Xingyi	56	60	45	0	161

^a^A chlorophyll meter was used to guide N application at panicle initiation and spikelet differentiation. SPAD was measured on the 10 topmost fully expanded leaves per plot as described by Peng et al. (1993). At panicle initiation, if SPAD < 37, apply 60 kg ha^−1^; if between 37 and 39, apply 45 kg ha^−1^; if > 39, apply 30 kg ha^−1^. At spikelet differentiation, if SPAD < 37, apply 45 kg ha^−1^; if between 37 and 39, apply 30 kg ha^−1^; if between 39 and 42, apply 15 kg ha^−1^; if > 42, apply 0 kg ha^−1^

Pre-germinated seeds were sown at a rate of 25 g m^−2^ on 10 March in Huaiji, 20 March in Binyang, 15 January in Haikou, 13 May in Changsha and 8 April in Xingyi. Seedlings were transplanted at a hill spacing of 20 cm × 27 cm with two seedlings per hill. Seedling age at transplanting was 27–33 d in Huaiji, 21–22 d in Binyang, 33–36 d in Haikou, 27 d in Changsha and 35 d in Xingyi. Phosphorus (112.5 kg P_2_O_5_ ha^−1^) was applied and incorporated in all subplots 1 day before transplanting. Potassium (157.5 kg K_2_O ha^−1^) was split equally at baseline and panicle initiation. The regimen for water management was in the sequence of flooding, midseason drainage, re-flooding and moist intermittent irrigation. Weeds, insects and diseases were intensively controlled with chemicals to avoid yield loss.

### Sampling and Measurements

Six hills were sampled in each subplot at full heading (approximately 80 % of the panicles had emerged from the flag leaf sheath, HD) and ten hills were diagonally sampled in each subplot at maturity (MA). For all the sampling procedures, the 3 border lines were excluded to avoid border effects. Plants sampled at HD were separated into stems, leaves and panicles. The dry weight of each organ was determined after oven-drying at 70 °C to constant weight. Plants sampled at MA were hand threshed after the panicles were counted. Filled spikelets were separated from unfilled spikelets by submerging them in tap water. Three subsamples of 30 g filled grains and all unfilled spikelets were taken to count the numbers of spikelets. The dry weights of straw (including rachis) and filled and unfilled spikelets were determined after oven drying at 70 °C to constant weight. CGR from sowing (SO) to HD and HD to MA, spikelets per panicle, spikelet filling percentage (100 × filled spikelet number/total spikelet number), and harvest index (100 × filled spikelet weight/aboveground total dry weight) were calculated. Grain yield was determined from a 5-m^2^ area in the middle of each subplot and adjusted to a moisture content of 0.14 g H_2_O g^−1^ fresh weight.

Daily minimum and maximum temperature data were obtained from the local meteorological bureaus. Daily solar radiation was calculated with a modified form of the Hargreaves-Samani model (Hargreaves and Samani 1982; Abraha and Savage 2008), and the estimated solar radiation agreed well with the measured values over a wide geographical region without the need for further site-specific calibration (Ball et al. 2004).

### Statistical Analysis

Data were analyzed using analysis of variance (Statistix 8, Analytical Software, Tallahassee, FL, USA). The means of cultivars and sites were compared based on the least significant difference test (LSD) at the 0.05 probability level.
